# Dual‐Driver Myeloproliferative Neoplasm: Concurrent p190 *BCR::ABL1* CML and *JAK2* V617F Mutation: A Case Report and Literature Review

**DOI:** 10.1155/crh/2927231

**Published:** 2026-09-22

**Authors:** Mohamed Elkady, Yulan Jin, Weiqiang Zhao

**Affiliations:** ^1^ Department of Pathology, Division of Hematopathology, The Ohio State University Wexner Medical Center, Columbus, Ohio, USA, osu.edu

**Keywords:** *BCR::ABL1*, chronic myeloid leukemia, dual-driver myeloproliferative neoplasm, e1a2 transcript, *JAK2* V617F, megakaryocyte morphology, p190 isoform

## Abstract

The concurrent occurrence of *BCR::ABL1* rearrangement and *JAK2* V617F mutation is an exceptionally rare phenomenon, with reported frequencies of 0.2%–2.5% in tested myeloproliferative neoplasm (MPN) cohorts and an estimated overall rate of approximately 0.4% in patients screened for both alterations. These molecular drivers are generally considered mutually exclusive, as *BCR::ABL1* defines Philadelphia chromosome‐positive (Ph+) chronic myeloid leukemia (CML), while *JAK2* V617F characterizes Ph‐negative MPNs. We report a male patient in his 50s with a previously detected *JAK2* V617F mutation, initially managed as essential thrombocythemia, who later presented with leukocytosis, macrocytic anemia, thrombocytopenia, and absolute monocytosis. Bone marrow demonstrated marked myeloid hyperplasia with atypical megakaryocytes, comprising scattered dwarf forms together with larger forms with “cloud‐like” nuclei and focal reticulin fibrosis (MF‐1). These findings, together with the known *JAK2* V617F mutation, prompted targeted molecular investigation. High‐sensitivity RT‐PCR confirmed an isolated e1a2 (p190) *BCR::ABL1* transcript, the minor breakpoint cluster region isoform occurring in only 1%‐2% of CML cases, with the complete absence of the p210 isoform, alongside a concurrent *JAK2* V617F mutation (allele burden 16%). Standard p210‐focused quantitative assays would have produced a false‐negative result in this case. The patient received first‐line asciminib and was subsequently transitioned to dasatinib, with the *BCR::ABL1*/*ABL1* ratio declining from 88.5% at diagnosis to 0.79% at most recent assessment. This case highlights the critical importance of integrated morphologic, cytogenetic, and molecular evaluation in atypical MPN presentations. The admixed megakaryocytic atypia superimposed on myeloid hyperplasia reflected the coexistence of two drivers. Comprehensive profiling, including high‐sensitivity RT‐PCR capable of detecting the p190 isoform and concurrent *JAK2* testing, is essential for accurate diagnosis, prognostication, and therapy selection in these rare dual‐driver cases.

## 1. Introduction

The diagnostic classification of myeloproliferative neoplasms (MPNs) has historically been governed by a principle of genetic exclusivity, wherein detection of the *BCR::ABL1* translocation serves as the defining marker for CML, while mutations in *JAK2*, *CALR*, or *MPL* characterize the Philadelphia chromosome‐negative (Ph−) MPNs such as polycythemia vera (PV), essential thrombocythemia (ET), and primary myelofibrosis (PMF) [1, 2]. However, advancements in high‐sensitivity molecular diagnostics and comprehensive bone marrow histopathology have identified a rare but clinically significant subset of “dual‐driver” MPNs in which both driver categories coexist [3].

The p190 *BCR::ABL1* (e1a2) isoform results from a minor breakpoint cluster region (*m*‐bcr) rearrangement and is rare in CML, occurring as the sole transcript in approximately 1%‐2% of cases [4, 5]. It is far more characteristic of Ph+ B‐lymphoblastic leukemia/lymphoma, particularly in the pediatric population [6]. Structurally, the p190 protein lacks the Dbl‐homology (DH) and pleckstrin‐homology (PH) domains present in p210, resulting in a hyperactive kinase state, a distinct interactome that preferentially activates Src family kinases such as Lyn, and a propensity for monocytic expansion that can resemble chronic myelomonocytic leukemia (CMML) [6, 7].

Coexistence of *BCR::ABL1* rearrangement with *JAK2* V617F is exceptionally uncommon [3]. Dual‐driver cases create significant diagnostic challenges due to overlapping or confounding morphologic features and variable clonal dynamics; one driver may predominate initially, with the other emerging or expanding under therapeutic pressure, a phenomenon termed “opposite growth” [8, 9]. Furthermore, the concurrent presence of *JAK2* V617F may be morphologically “masked” by a dominant CML clone, only to be revealed by specific megakaryocytic atypia such as cloud‐like nuclei and tight clustering [8]. This report details a case of concurrent p190 *BCR::ABL1* CML and *JAK2* V617F, emphasizing integrated morphologic, cytogenetic, and molecular evaluation and discusses the clinical and therapeutic implications of this rare dual‐driver scenario.

## 2. Case Presentation

### 2.1. Clinical Presentation

This case was identified and managed at the authors’ tertiary academic medical center. A male patient in his 50s with a previously detected *JAK2* V617F mutation, initially managed as low‐risk ET with aspirin, presented approximately 14 months later with chronic fatigue and modest weight loss. His medical history included coronary artery disease with prior myocardial infarction and percutaneous coronary intervention. He denied lymphadenopathy or bone pain. Physical examination demonstrated splenomegaly, without hepatomegaly or lymphadenopathy.

Initial complete blood count demonstrated marked leukocytosis (white blood cell count 65.1 × 10^9^/L; reference 4.5–11.0), with absolute monocytosis (8.5 × 10^9^/L). There was a moderate macrocytic normochromic anemia (hemoglobin 10.0 g/dL; mean cell volume 102.3 fL) with anisocytosis, and mild thrombocytopenia (platelets 94 × 10^9^/L). The manual differential revealed a marked left shift (blasts 1%, promyelocytes 1%, myelocytes 6%, metamyelocytes 11%, neutrophils 56%, lymphocytes 4%, monocytes 13%, eosinophils 2%, and basophils 6%). Peripheral blood smear confirmed leukocytosis with rare circulating blasts and left‐shifted granulocytosis; Auer rods were not identified. Red cells showed rare teardrop forms and hypochromasia, and platelet morphology was normal. The prominent monocytosis at presentation was a clinically notable feature consistent with the p190 *BCR::ABL1* phenotype [10].

### 2.2. Bone Marrow Pathology

**FIGURE 1:**
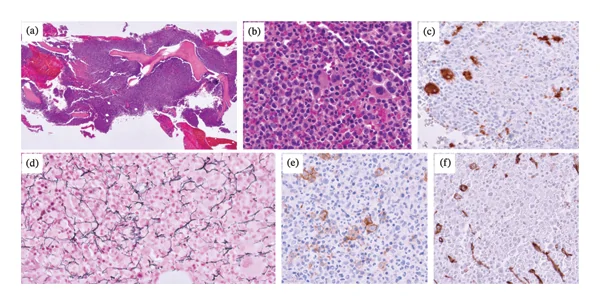
Bone marrow biopsy findings in dual‐driver p190 *BCR::ABL1*/*JAK2* V617F myeloproliferative neoplasm. (a) H&E, low power: markedly hypercellular marrow (> 95%) with myeloid hyperplasia and left shift; myeloid:erythroid ratio 7:1. (b) H&E, 400x: atypical megakaryocytes comprising scattered dwarf forms together with larger, pleomorphic forms with bulbous “cloud‐like” nuclei, some in loose aggregates. (c) CD61 immunohistochemistry stain, 400x, highlights the megakaryocytes and confirms their atypia and abnormal distribution, including a subset of small/dwarf forms, without well‐formed dense clusters. (d) Reticulin silver stain, 400x: Focal MF‐1 fibrosis with coarse intersecting fibers; consistent with a *JAK2*‐driven myelofibrotic component. (e) E‐cadherin immunohistochemistry stain, 400x, highlights erythroid precursors and their intramedullary distribution. (f) CD34 immunohistochemistry, 400x: Scattered blasts comprising 2%‐3% of marrow cellularity; blast crisis excluded.

Histopathologic assessment included hematoxylin‐and‐eosin (H&E)–stained core biopsy and aspirate smears, reticulin staining graded per WHO 5th Edition and ICC 2022 criteria [1, 2], and immunohistochemistry (IHC) for CD61, CD34, and E‐cadherin. The bone marrow biopsy was markedly hypercellular (> 95%) with marked myeloid hyperplasia and left shift, yielding a myeloid:erythroid ratio of 7:1 and preserved granulocytic maturation through all stages (Figure 1a). Megakaryocytes were atypical, comprising a mixture of forms including scattered dwarf megakaryocytes together with larger, pleomorphic forms with bulbous, “cloud‐like” nuclei, some in loose aggregates (Figure 1b). These megakaryocytic features are more characteristic of *JAK2*‐driven Ph‐negative MPNs than classic p210 CML, in which megakaryocytes are typically small, hypolobated “dwarf” forms without significant clustering [11, 12]. The presence of these atypical megakaryocytic forms in a Ph+ marrow, together with the patient’s known *JAK2* V617F mutation, supported a dual‐driver process [8, 13].

CD61 IHC highlighted the megakaryocytes and confirmed their atypia and abnormal distribution, including a subset of small/dwarf forms, without well‐formed dense clusters (Figure 1c). Reticulin silver stain demonstrated focal MF‐1 fibrosis with coarse intersecting fibers, consistent with a *JAK2*‐driven myelofibrotic component (Figure 1d) [1]. E‐cadherin highlighted erythroid precursors (Figure 1e), and CD34 highlighted scattered blasts (2%‐3% of marrow cellularity) (Figure 1f), excluding blast crisis. Flow cytometry immunophenotyping confirmed no increase in CD34+ or TdT+ blasts, excluding concurrent Ph+ B‐lymphoblastic leukemia/lymphoma [6].

### 2.3. Cytogenetic and Molecular Findings

**FIGURE 2:**
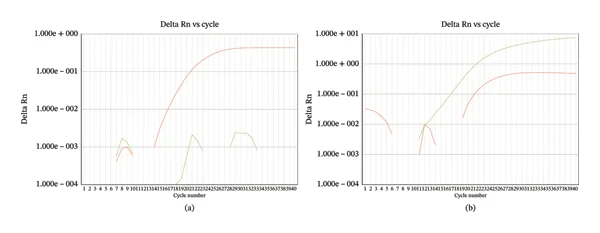
Qualitative RT‐PCR amplification plots (delta Rn vs. cycle number) confirming an isolated p190 *BCR::ABL1* transcript. Panels are native high‐resolution exports from the instrument software; the two panels retain their original instrument *y*‐axis ranges. (a) p210 *BCR::ABL1*‐specific assay: Positive control curve (red) demonstrates assay validity; no patient‐level amplification above threshold confirms the complete absence of the p210 (e13a2/e14a2) isoform. Standard p210‐focused assays would have produced a false‐negative result in this case. (b) p190 *BCR::ABL1*‐specific assay: Robust sigmoidal amplification in the patient sample (threshold crossing Ct ∼12), confirming exclusive e1a2 minor breakpoint transcript expression.

Conventional G‐banded karyotyping revealed the Philadelphia chromosome: 46, XY, *t*(9; 22) (q34; q11.2) [14]. FISH using a dual‐color, dual‐fusion *BCR::ABL1* probe confirmed the translocation in 93.5% of nuclei. High‐sensitivity qualitative RT‐PCR confirmed an isolated e1a2 (p190) *BCR::ABL1* transcript without any detectable p210 (e13a2 or e14a2) isoform (Figure 2) [5, 10]. The p210‐specific assay demonstrated valid positive‐control amplification (Ct ∼14) without patient‐level signal (Figure 2a), while the p190‐specific assay yielded robust amplification (Ct ∼12), confirming exclusive e1a2 transcript expression (Figure 2b). This finding is of critical diagnostic importance, as standard p210‐focused quantitative assays may fail to detect the p190 variant, potentially leading to missed or delayed diagnosis [5].

Concurrent pyrosequencing identified the *JAK2* V617F mutation with an allele burden of 16%. The *BCR::ABL1*/*ABL1* ratio at diagnosis was 88.5%, while p210 transcripts were undetectable. *ABL1* kinase domain mutational analysis revealed no resistance mutations.

### 2.4. Treatment and Clinical Course

First‐line therapy was asciminib 80 mg once daily, a STAMP (Specifically Targeting the ABL Myristoyl Pocket) allosteric inhibitor, begun 2 weeks after diagnosis [15]. Asciminib was selected in preference to a second‐generation tyrosine kinase inhibitor (TKI) because of the patient’s cardiovascular comorbidity, which argues against agents carrying appreciable arterial occlusive or cardiopulmonary risk. On asciminib, the *BCR::ABL1*/*ABL1* ratio fell from 88.5% at diagnosis to 21.2%, but subsequently rose to 29.7% and then 53.0% at approximately 2 months. As this rise occurred despite documented adherence and *ABL1* kinase domain sequencing showed no resistance mutation, therapy was changed. The patient was transitioned to dasatinib 140 mg once daily at approximately 3 months. Dasatinib was preferred over imatinib, bosutinib, or nilotinib because it is a potent second‐generation ATP‐competitive inhibitor with broad activity across most kinase domain mutations, is administered once daily, and additionally inhibits Src family kinases including Lyn, through which p190 *BCR::ABL1* preferentially signals [16]. A sample drawn immediately before the switch subsequently resulted at 5.95%, indicating that a delayed response to asciminib had begun; this result became available only after the transition had been made. On dasatinib, the molecular response continued to improve, with serial ratios of 4.07%, 4.58%, 1.66%, and most recently 0.79% at approximately 9 months from initiation of TKI therapy. This most recent value lies below the 1% threshold but has not yet reached major molecular response (0.1%), and quantitative monitoring continues at three‐monthly intervals. Hematologic parameters normalized in parallel, with the white blood cell count returning to the reference range, hemoglobin improving to 12.3‐12.4 g/dL, and the platelet count falling from 708 to 550 × 10^9^/L. Should an adequate and durable molecular response not be sustained, allogeneic hematopoietic stem cell transplantation remains a definitive option in this dual‐driver setting [17].

## 3. Discussion

### 3.1. Molecular Biology of the p190 *BCR::ABL1* Isoform

The *BCR::ABL1* fusion gene results from the *t*(9; 22) (q34; q11.2) translocation. While the *ABL1* breakpoint is generally constant, the BCR breakpoint varies across three principal cluster regions, each conferring a distinct oncoprotein size, domain composition, and clinical phenotype (Table 1) [18]. The p190 isoform lacks the DH and PH domains present in p210, yielding a hyperactive kinase that preferentially signals through Src family kinases (particularly Lyn), RAS/MAPK, and PI3K/AKT/mTOR pathways [6]. This distinct signaling landscape underlies the clinical phenotype of p190 CML, which characteristically includes peripheral monocytosis mimicking CMML and has been associated with poorer initial responses to imatinib [4, 5, 7, 10]. Notably, the p190 isoform appears disproportionately represented among concurrent *BCR::ABL1* and *JAK2* V617F cases relative to its overall frequency in CML [3, 5].

**TABLE 1:** *BCR::ABL1* fusion transcript types and associated clinical phenotypes.

BCR breakpoint	Transcript	Protein	Associated phenotype
Major (M‐bcr)	e13a2/e14a2	p210	Classic CML; ∼30% adult ALL
Minor (m‐bcr)	e1a2	p190	∼75% pediatric Ph+ ALL; rare CML (1%–2%)
Micro (μ‐bcr)	e19a2	p230	Chronic neutrophilic leukemia

Abbreviations: ALL, acute lymphoblastic leukemia; CML, chronic myeloid leukemia.

### 3.2. Pathophysiology of Concurrent *JAK2* V617F

The *JAK2* V617F mutation results from a valine‐to‐phenylalanine substitution at Codon 617, disrupting the pseudokinase (JH2) autoinhibitory domain and leading to constitutive activation of the JAK‐STAT axis, particularly STAT5, which drives trilineage myeloid proliferation [19]. The most prevalent hypothesis is that concurrent cases represent two distinct or partially overlapping clones (biclonal or composite disease) arising from a common hematopoietic stem cell or independently, rather than a single clone harboring both events [3]. While TKI therapy suppresses *BCR::ABL1*‐mediated signaling, the *JAK2* V617F clone continues to provide STAT5‐mediated survival signals, creating a potential escape mechanism during TKI monotherapy [6, 19]. This underscores the necessity of monitoring both drivers independently throughout treatment.

### 3.3. Histomorphologic Clues to Dual Pathology

Megakaryocytic morphology provides essential diagnostic information in MPN classification and serves as a critical clue in dual‐driver cases (Table 2) [1, 11]. In classic p210 CML, megakaryocytes are characteristically small (“dwarf” forms) with hypolobated nuclei and dispersed distribution; in contrast, *JAK2* V617F‐driven MPNs with a myelofibrotic component display large megakaryocytes with “cloud‐like” bulbous nuclei and dense paratrabecular clustering [1, 12]. In the present case, these atypical features superimposed on a CML‐like myeloid hyperplasia provided the key morphologic clue to dual pathology [8, 12]. CD61 IHC confirmed the clustering and abnormal distribution, and concurrent focal reticulin fibrosis (MF‐1) further supported a *JAK2*‐driven myelofibrotic component [11, 12].

**TABLE 2:** Megakaryocytic morphology in myeloproliferative neoplasms by driver mutation.

MPN driver	Megakaryocyte size	Nuclear feature	Topography
*BCR::ABL1* (p210)	Small (dwarf)	Hypolobated	Dispersed
*BCR::ABL1* (p190)	Small–intermediate	Hypolobated/atypical	Dispersed ± monocytosis
*JAK2* V617F (PV/ET)	Medium to large	Pleomorphic/bulbous	Loose clusters
*JAK2* V617F (PMF)	Variable	Cloud‐like/bulbous	Tight clusters
CALR (PMF/ET)	Large to giant	Staghorn‐like	Tight clusters

*Note:* PMF: primary myelofibrosis.

Abbreviations: ET, essential thrombocythemia; PV, polycythemia vera.

### 3.4. Therapeutic Considerations and Monitoring

Our patient’s transcript levels fluctuated on asciminib before transition to dasatinib, after which the response improved. We caution against overinterpreting this sequence: there is at present no published clinical evidence that asciminib efficacy differs systematically between the p210 and p190 isoforms, and asciminib trials have not reported isoform‐stratified outcomes. Any biological rationale, such as the reliance of p190 on Src family signaling that dasatinib but not asciminib targets, remains speculative and requires formal study [15, 16, 20, 21]. Managing a dual‐driver MPN requires targeting both oncogenic clones to prevent “opposite growth,” wherein suppression of one clone permits outgrowth of the other, documented in up to 81.8% of concomitantly treated cases in the largest systematic review [8, 9]. Serial quantitative monitoring of both *BCR::ABL1* (by e1a2‐specific assay) and *JAK2* V617F allele burden is, therefore, mandatory throughout therapy [8]. The combination of TKI and JAK1/2 inhibition (ruxolitinib) has been successfully employed in select reported dual‐driver cases and may be considered when *JAK2*‐driven progression is detected [22]. Standard clinical workflows for *BCR::ABL1* quantification utilize p210‐specific assays that may fail entirely to detect the p190 isoform; high‐sensitivity e1a2‐specific RT‐qPCR must be explicitly requested when p190 CML is suspected, particularly with monocytosis or atypical megakaryocytic morphology [10].

### 3.5. Comparative Literature Review

Several case series and institutional cohorts have documented the concurrent presence of *BCR::ABL1* and *JAK2* V617F (Table 3) [3, 8, 9]. The largest multi‐institutional series by Soderquist et al. described 11 such patients, establishing a frequency of approximately 0.4% in tested MPNs; notably, 3 of 11 cases harbored the p190/e1a2 isoform, suggesting enrichment of this rare subtype in the dual‐driver setting [3]. Zanelli et al. documented the “opposite growth” phenomenon in their larger cohort [8]. We acknowledge that this combination, including instances involving the p190 isoform, is not itself unprecedented: In addition to the series of Soderquist et al. [3] and the review of Zanelli et al. [8], Caocci et al. reported an early individual case of simultaneous p190 *BCR::ABL1* and *JAK2* V617F responding to imatinib [24]. The particular contribution of the present report is its integrated histopathologic emphasis, its documentation of the false‐negative risk of p210‐directed assays in this setting, and its detailed real‐world sequential‐TKI course. The 2022 WHO 5th edition and ICC classifications do not formally define the “dual‐driver” entity, representing a gap in current nosology [1, 2].

**TABLE 3:** Summary of key published cases and series of concurrent *BCR::ABL1* and *JAK2* V617F myeloproliferative neoplasms.

Reference/year	Key findings	Cases/focus	Relevance
Soderquist et al. [3], 2018	Largest series; frequency ∼0.4%; often composite (two clones); higher‐than‐expected p190 proportion (3/11); morphology can be hybrid	11 (3 p190)	p190 overrepresentation; morphologic overlap; clonal dynamics
Zanelli et al. [8], 2024	Co‐occurrence rare (0.2%–2.5%); “opposite growth” in 81.8% of concomitantly treated patients	87 (review)	Dual‐driver context; treatment‐response confounding
Soriani et al. [14], 2022	Concurrent *JAK2* V617F evolving with CML (rare e1a2/p190); phenotype shifted with treatment	Single case	Exact molecular profile; treatment‐dependent phenotype
Bader and Dreiling [23], 2019	Review of 33 published *JAK2*+ MPN + CML cases (mostly p210); variable clonal hierarchy	Review (33)	Broad dual‐driver context and management
Caocci et al. [24], 2010	CML with simultaneous p190 *BCR::ABL1* + *JAK2* V617F; imatinib response documented	Single case	Early specific p190 + *JAK2* report
De Roeck et al. [25], 2018	Under TKI, *BCR::ABL1* suppression can lead to emergence/expansion of *JAK2* clone	Series (11)	Clonal dynamics; treatment implications
Adnan‐Awad et al. [26], 2021	p190 CML: poorer initial imatinib response, more epigenetic mutations, distinct signaling, monocytosis	Multiple p190	p190‐specific biology and TKI response
Martin‐Cabrera et al. [27], 2017	23 cases; combined drivers associated with inferior prognosis	23 cases	JAK/STAT resistance pathway
Paciaroni et al. [22], 2025	Concurrent CML and *JAK2*+ myelofibrosis treated with TKI + ruxolitinib	Single case	Supports combination therapy approach
Gagnon et al. [28], 2026	Large cohort; dual‐driven MPNs associated with TKI resistance and inferior survival	Multicenter cohort	Risk stratification; early therapeutic failure

*Note:* MPN: myeloproliferative neoplasm.

Abbreviations: CML, chronic myeloid leukemia; TKI, tyrosine kinase inhibitor.

## 4. Conclusion

This case exemplifies the diagnostic and therapeutic complexity inherent to true dual‐driver MPNs harboring concurrent p190 *BCR::ABL1* and *JAK2* V617F. The atypical megakaryocytic morphology, comprising admixed dwarf and larger pleomorphic forms with cloud‐like nuclei, superimposed on a CML‐like myeloid hyperplasia, reflected the coexistence of two drivers, and reinforced the value of correlating morphology with molecular findings [8, 11]. Comprehensive molecular profiling, including high‐sensitivity RT‐PCR capable of detecting the p190 isoform and reflex *JAK2* testing when morphology is discordant with classic CML, is essential in all atypical MPN presentations [5, 10]. The fluctuating response to first‐line asciminib and subsequent improvement on dasatinib should be interpreted cautiously, as no isoform‐specific difference in asciminib efficacy has been established; TKI selection should rest on resistance testing, comorbidity, and tolerability [15, 16]. Serial comonitoring of both *BCR::ABL1* and *JAK2* allele burden is mandatory to detect “opposite growth” and guide timely therapeutic modifications, potentially including the addition of ruxolitinib [8, 9, 22]. Early recognition of this rare dual‐driver entity is essential to prevent misattribution of treatment responses, avoid diagnostic pitfalls, and optimize patient outcomes through precision‐guided hematologic management.

## Author Contributions

Mohamed Elkady contributed to conceptualization, case identification, data acquisition, literature review, manuscript drafting, and figure preparation. Yulan Jin contributed to case evaluation, data interpretation, and critical manuscript revision. Weiqiang Zhao contributed to senior supervision, case sign‐out, molecular diagnostic oversight, critical manuscript revision, and final approval.

## Funding

This study was not supported by any funding.

## Disclosure

All authors have read and approved the final version of the manuscript. Weiqiang Zhao (corresponding author) had full access to all of the data in this study and takes complete responsibility for the integrity of the data and the accuracy of the data analysis.

## Ethics Statement

This study was conducted in accordance with the ethical standards of the institutional research committee and with the 1964 Helsinki Declaration and its later amendments. Institutional Review Board approval was obtained from The Ohio State University Wexner Medical Center. The IRB determined that individual informed consent was not required given the retrospective, deidentified nature of the case report.

## Consent

Please see the Ethics Statement.

## Conflicts of Interest

The authors declare no conflicts of interest.

## Supporting Information

Additional supporting information can be found online in the Supporting Information section.

## Supporting information


**Supporting Information** This case report was prepared in accordance with the CAse Report (CARE) guidelines; a completed CARE checklist is provided as supporting information.

## Data Availability

All relevant data supporting the findings of this case report are included within the article. Additional deidentified data are available from the corresponding author upon reasonable request.
