# Supplementary material for: Dual‐Driver Myeloproliferative Neoplasm: Concurrent p190 BCR::ABL1 CML and JAK2 V617F Mutation: A Case Report and Literature Review
Source: Case Rep Hematol. 2026 Sep 22;2026:2927231. doi: 10.1155/crh/2927231 (PMC13598437; doi:10.1155/crh/2927231)
Supplement: Supplementary file 1 [file CRH-2026-2927231-s001.docx]

**CARE Checklist (2016), Completed**

*Manuscript 2927231, Dual-Driver Myeloproliferative Neoplasm (p190 BCR::ABL1 / JAK2 V617F)*

| **Item** | **CARE Checklist Topic** | **Description / Location in Manuscript** | **Reported (Line No.)** |
| --- | --- | --- | --- |
| 1 | Title | The words "case report" and the key diagnosis appear in the title | **Lines 1–2** |
| 2 | Key Words | Seven key words including *BCR::ABL1*, *JAK2* V617F, p190 isoform | **Line 35** |
| 3a | Abstract – Introduction | Rarity of dual-driver MPN; what is unique | **Lines 11–34** |
| 3b | Abstract – Case summary | Presentation, findings, diagnosis, treatment, outcome | **Lines 11–34** |
| 3c | Abstract – Conclusion | Reflex testing for a second driver when features are discordant | **Lines 11–34** |
| 4 | Introduction | Background with references to medical literature | **Lines 37–62** |
| 5a | Patient Information – Demographics | Male patient in his 50s | **Lines 63–80** |
| 5b | Patient Information – Main concerns/symptoms | Chronic fatigue and modest weight loss | **Lines 63–80** |
| 5c | Patient Information – Medical/family history | Prior *JAK2* V617F-mutated essential thrombocythemia on aspirin | **Lines 63–80** |
| 5d | Patient Information – Relevant comorbidities | Coronary artery disease with prior MI and PCI | **Lines 63–80** |
| 6 | Clinical Findings | Splenomegaly on examination; no lymphadenopathy | **Lines 63–80** |
| 7 | Timeline | *JAK2* detected, CML diagnosed ~14 months later; serial follow-up | **Lines 63–136** |
| 8a | Diagnostic – Methods | CBC, marrow biopsy, IHC, karyotype, FISH, RT-PCR, pyrosequencing (Figures 1–2) | **Lines 81–113** |
| 8b | Diagnostic – Challenges | p210 assay false-negative risk; morphologic ambiguity | **Lines 101–113, 173–189** |
| 8c | Diagnostic – Reasoning | Correlation of marrow morphology with dual molecular findings | **Lines 81–100, 161–172** |
| 8d | Diagnostic – Prognostic characteristics | Dual-driver biology; fluctuating then improving molecular response | **Lines 114–136, 173–189** |
| 9a | Therapeutic – Types of intervention | Asciminib then dasatinib | **Lines 114–136** |
| 9b | Therapeutic – Administration | Asciminib 80 mg daily; dasatinib 140 mg daily | **Lines 114–136** |
| 9c | Therapeutic – Changes | Switch from asciminib to dasatinib with rationale | **Lines 114–136** |
| 10a | Follow-up – Outcomes | Counts normalized; *BCR::ABL1* ratio 88.5% at diagnosis → 0.79% at ~9 months | **Lines 114–136** |
| 10b | Follow-up – Assessment | Serial CBC and molecular monitoring | **Lines 114–136** |
| 10c | Follow-up – Adherence/tolerability | Adherence documented; no reported treatment intolerance | **Lines 114–136** |
| 10d | Follow-up – Adverse events | No treatment-limiting adverse events reported | **Lines 114–136** |
| 11a | Discussion – Strengths/limitations | Integrated morphologic–molecular approach; single case | **Lines 138–206** |
| 11b | Discussion – Literature | Comparative review with Table 3 | **Lines 190–206** |
| 11c | Discussion – Rationale for conclusions | Morphologic–molecular correlation in dual-driver disease | **Lines 207–223** |
| 11d | Discussion – Takeaway | Reflex *JAK2* + isoform-specific RT-PCR | **Lines 207–223** |
| 12 | Patient Perspective | Not obtained; consent waived under approved IRB protocol | **N/A** |
| 13 | Informed Consent | IRB waiver of consent; no identifying data | **Lines 224–255** |

*This completed CARE checklist is submitted as supplementary material in support of the revised manuscript.*
